# Circulating Levels of Bone and Inflammatory Markers in Gestational Diabetes Mellitus

**DOI:** 10.1089/biores.2018.0013

**Published:** 2018-08-01

**Authors:** Deirdre Cocks Eschler, Georgia Kulina, Adolfo Garcia-Ocana, Jiawen Li, Thomas Kraus, Carol J. Levy

**Affiliations:** ^1^ Division of Endocrinology and Metabolism, Stony Brook University Hospital, Stony Brook, New York.; ^2^ Harbor View Medical Services, Division of Endocrinology, Mather Hospital Northwell Health, Port Jefferson, New York.; ^3^ Division of Endocrinology Diabetes and Bone Disease, Icahn School of Medicine at Mount Sinai, New York, New York.; ^4^ Department of Population Health Science & Policy, Icahn School of Medicine at Mount Sinai, New York, New York.; ^5^ Department of Center for Therapeutic Antibody Development, Icahn School of Medicine at Mount Sinai, New York, New York.

**Keywords:** gestational diabetes mellitus, hepatocyte growth factor, osteoprotegerin, RANK ligand

## Abstract

Gestational diabetes mellitus (GDM) can cause short- and long-term complications to the mother and fetus. While the precise mechanisms in preserving glucose balance in a healthy pregnancy are unknown, various growth factors and hormones have been implicated or associated with GDM risk in humans or rodents, including prolactin, tumor necrosis factor alpha (TNFα), osteoprotegerin (OPG), hepatocyte growth factor (HGF), and receptor activator of nuclear factor-kappa B ligand (RANKL). We aimed to evaluate the relationship of these and other protein markers in women with GDM. In this cross-sectional study, blood samples were collected from pregnant women with GDM and with normal glucose tolerance (NGT) at the 24- to 32-week obstetrical visit, during the 1-h oral glucose challenge test or 3-h oral glucose tolerance test. Blood plasma was analyzed for RANKL, OPG, prolactin, tumor necrosis factor-related apoptosis-inducing ligand (TRAIL), HGF, plasminogen activator inhibitor type 1 (PAI-1), and TNFα. Forty-six women with NGT and 47 women with GDM were included (mean ± standard deviation maternal age 31.6 ± 5.7, mean ± standard deviation gestational age 28.1 ± 2.2 weeks). Groups were similar in terms of age, body mass index, gestational age, and race/ethnicity. Serum levels of OPG, prolactin, TRAIL, HGF, PAI-1, and TNFα were similar in both groups. RANKL was lower in GDM subjects (*p* = 0.019). Contrary to previous reports in the literature, we found a lower serum RANKL level in women with GDM. Further investigation is needed to determine whether there are suitable serum markers for diagnosing GDM or determining prognosis or severity.

## Introduction

Gestational diabetes mellitus (GDM) is a complication of pregnancy with short-term and long-term maternal and fetal consequences. The incidence of GDM is increasing worldwide, with some reports affecting as high as 10% of pregnancies.^1,2^

GDM is thought to develop as a result of the inability of pancreatic β cells to overcome the natural insulin resistance created by placental hormones in the second half of pregnancy as well as a loss of first phase insulin secretion.^3^ Healthy pregnant women adapt to this demand by increasing insulin secretion through β cell hyperplasia and hypertrophy and, as shown in animal models, when this fails to occur, GDM ensues.^4–7^

Pituitary and placental lactogens are known to play a role in the ability of healthy pregnant women to overcome the increased insulin demands, but the exact molecular mechanisms by which this occurs have yet to be fully elucidated.^8,9^ Alterations in inflammatory markers, bone metabolism, and growth factor signaling have been implicated in abnormal glucose tolerance and other diseases in pregnant women.^10–12^ For example, osteoprotegerin (OPG) and receptor activator of nuclear factor-kappa B ligand (RANKL), cytokines thought to be predominantly involved in bone metabolism, have shown altered ratios in patients with GDM.^12^ Also, tumor necrosis factor-related apoptosis-inducing ligand (TRAIL), an important factor in apoptosis, has been found to circulate at lower levels in women with hypertensive diseases, whereas elevated plasma levels of plasminogen activator inhibitor type 1 (PAI-1) have been associated with insulin resistance and diabetes.^13^

Hepatocyte growth factor (HGF), a growth factor produced by liver and placental tissues that promotes cell survival and tissue regeneration, has been implicated in mouse models of gestational diabetes.^11,14^ Pregnant mice lacking the HGF receptor, known as c-Met, demonstrate higher blood glucose values and lower plasma insulin levels.^11^ Additionally, circulating levels of tumor necrosis factor alpha (TNFα), have been found to be higher in women with GDM.^15–17^

The purpose of this study was to further evaluate the relationship of various protein markers, including prolactin, HGF, TNFα, TRAIL, PAI-1, RANKL, and OPG, with gestational diabetes in women.

## Materials and Methods

**FIG. 1.**
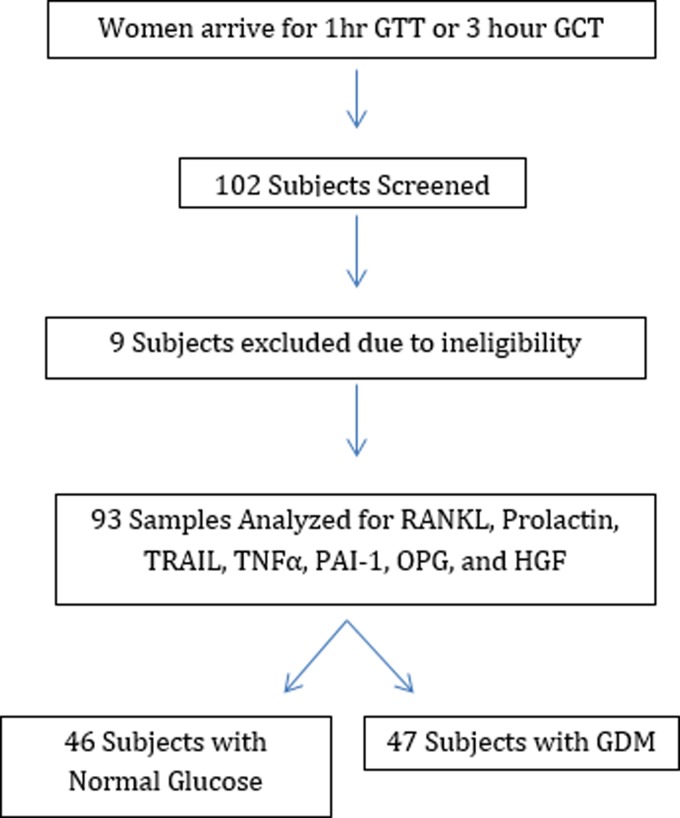
Subject selection. Nine subjects were ineligible due to maternal age outside eligibility window, subjects not in appropriate gestational window, and missing subject data needed to confirm eligibility.

This was a two-site, cross-sectional study. A total 102 women who were between 24 and 32 weeks pregnant were consented and recruited for the study at the obstetric clinics at the Icahn School of Medicine at Mount Sinai and Elmhurst Hospital Center in New York between October 3, 2013 and April 21, 2015. IRB (Institutional Review Board) approval was obtained and voluntary patient participation with informed consent occurred for all study subjects. Inclusion criteria were all adult pregnant patients >18 years of age who were between 24 and 32 weeks gestation. Exclusion criteria were pre-existing type 1 or type 2 diabetes mellitus. Patients were randomly invited to participate in the study during their routine obstetrics visits by our team. Ten milliliters of blood was collected from each patient during the screening of 1-h oral glucose challenge test (OGCT) or the 3-h oral glucose tolerance test (OGTT) between 24 and 32 weeks gestational age. Charts were subsequently reviewed and women who were sampled during the 1-h OGTT and had a 1 h glucose ≥135 mg/dL, received a 3-h OGTT. If the patients had a glucose value over 135 and failed to follow up with the 3-h OGTT, their samples were excluded from the analysis. GDM status was diagnosed based on the Carpenter and Costan criteria such that those who had two or more of the following were classified as having GDM; fasting glucose ≥95 mg/dL, 1-h glucose ≥180 mg/dL, 2-h glucose ≥155 mg/dL, and 3-h glucose ≥140 mg/dL. Patients were divided accordingly to GDM status. Forty-seven patients were diagnosed with GDM, and 46 patients were used as controls. Nine patients were excluded because they did not meet eligibility criteria (Fig. 1).

### Blood sampling

Venous blood samples (10 mL) were drawn in lavender top tubes with EDTA preservative during the 1-h OGCT or 3-h OGTT. Samples were obtained from women between 24 and 32 weeks of gestation. The samples were placed on ice immediately and centrifuged within 1 h of collection at 3000 *g*, 4°C for 15 min. Plasma was subsequently aliquoted separately and immediately stored at −80°C until assayed.

### Multiplex cytokine/chemokine/hormone assay of plasma HGF, RANKL, OPG, TRAIL, prolactin, TNFα, and PAI-1 (total) levels

Cytokines/chemokines/hormones levels in human plasma were assessed by Magnetic Bead-Based Multiplex Assays using the Luminex technology (EMD Millipore) following the manufacturer's instruction. Multiplex plates were read using a Luminex 100 multiplex plate reader (Luminex, Austin, TX).

### Statistical analyses

All results are presented as mean ± standard deviation for continuous covariates and frequencies and percentages for categorical variables. We used a two-way ANOVA for continuous variables and chi-squared tests for categorical variables to compare GDM and normal patients. A two-tailed significance level of 0.05 was used in all tests. Statistical analyses were performed using SAS software (SAS 9.4 Institute, Inc., Cary, NC). Due to the similarities between the groups at baseline, multivariate analysis was not performed.

### Clinical data collection

Following delivery, we performed a retrospective chart analysis in the electronic medical record to assess for pregnancies complicated by pre-eclampsia, small or large for gestational age babies, C-section delivery, neonatal hypoglycemia, early labor (<37 weeks), and maternal treatment for GDM.

## Results

Forty-six women with normal glucose tolerance (NGT) and 47 women with GDM were included in the study. There were no significant differences between subjects with GDM and those without in terms of maternal age, gestational age, body mass index (BMI), mean arterial pressure, and race/ethnicity (Table 1). There were no significant differences in daily medication use or past medical history. One subject in the GDM group had a history of GDM, and one subject in the GDM group had a history of hypothyroidism. No other endocrine abnormalities were reported in any of the other subjects. Treatment of GDM was determined by clinician preference and included diet (23 subjects), glyburide (24 subjects), or glyburide+insulin (one subject).

**Table 1.** **Patient Characteristics**

Patient characteristics	Over all (*n* = 93)	NGT (*n* = 46)	GDM (*n* = 47)	*p*
Clinical				
Maternal age, years	31.6 ± 5.7	31.48 ± 6.16	31.43 ± 4.12	0.865
Gestational age, weeks	28.1 ± 2.2	28.02 ± 1.71	28.69 ± 2.37	0.671
BMI	30.7 ± 6.6	30.4 ± 7.69	29.15 ± 4.71	0.643
MAP	81.0 ± 7.7	80.27 ± 7.66	81.2 ± 8.9	0.395
Race/Ethnicity, *n* (%)				0.518
Asian	17 (18.3)	6 (13.04)	11 (23.4)	
African American	13 (14.0)	8 (17.39)	5 (10.64)	
Caucasian/Non-Hispanic	13 (14.0)	8 (17.39)	5 (10.64)	
Hispanic	45 (48.4)	21 (45.65)	24 (51.06)	
Unknown	5 (5.4)	3 (6.52)	2 (4.26)	

BMI, body mass index; GDM, gestational diabetes mellitus; NGT, normal glucose tolerance.

Serum concentrations of HGF, OPG, TRAIL, prolactin, TNFα, and PAI-1 were not significantly different between those with NGT and those with GDM (Table 2). Serum RANKL was significantly lower in patients with GDM (40.15 ± 28.55 vs. 27.95 ± 27.59, *p* = 0.019).

**Table 2.** **Results of Protein Analysis for Women with Normal Glucose Tolerance and Women with Gestational Diabetes Mellitus**

	NGT	GDM	*p*
OPG, pg/mL	616.46 ± 279.02	624.11 ± 315.88	0.717
RANKL, pg/mL	40.15 ± 28.55	27.95 ± 27.59	0.019
TRAIL, pg/mL	110.84 ± 36.62	103.68 ± 41.4	0.238
Prolactin, ng/mL	259.61 ± 139.48	332.33 ± 173.31	0.127
HGF, pg/mL	118.47 ± 71.12	124.08 ± 69.21	0.652
TNFα, pg/mL	2.61 ± 1.29	2.74 ± 1.1	0.604
PAI-1 (total), ng/mL	632.01 ± 281.45	639.44 ± 312.51	0.904

HGF, hepatocyte growth factor; OPG, osteoprotegerin; PAI-1, plasminogen activator inhibitor type 1; RANKL, receptor activator of nuclear factor-kappa B ligand; TNFα, tumor necrosis factor alpha; TRAIL, tumor necrosis factor-related apoptosis-inducing ligand.

As has been previously demonstrated, there was a trend toward increased rates of C-section, macrosomia, and preterm delivery in patients with GDM (Table 3). Two patients in the NGT group and three patients in the gestational diabetes group did not have clinical outcomes data available. However, the results did not meet statistical significance as the study was not powered to detect differences in clinical outcomes. BMI was statistically significantly higher in individuals with pre-eclampsia compared with subjects with NGT (34.8 ± 9.7 vs. 30.4 ± 5.6, *p* = 0.021). Mean arterial pressure was also higher in individuals with pre-eclampsia compared with those without (86.7 ± 7.3 vs. 79.7 ± 7.4, *p* = 0.002).

**Table 3.** **Clinical Outcomes**

	NGT (*n* = 44)	GDM (*n* = 44)	*p*
Macrosomia (>4000 g)	2	5	0.435
Preterm delivery (before 37 weeks)	2	6	0.266
Pre-eclampsia	7	7	1
C-section	16	20	0.386

Clinical outcomes were obtained from 44 women with NGT and 44 women with GDM. Two patients in the NGT group and three patients in the gestational diabetes group did not have clinical outcomes data available.

## Discussion

In the present study, we found that serum RANKL was significantly lower in women with GDM compared with those with NGT, even when adjusted for age, BMI, and gestational age. However, no significant differences were observed for serum values of HGF, TRAIL, OPG, prolactin, TNFα, and PAI-1 when compared with controls.

GDM is a significant health concern that can lead to maternal and fetal complications. Universal screening of all pregnant women is recommended to identify and treat women with GDM in an effort to reduce complications and improve outcomes. Screening occurs at 24–28 weeks gestation in those without risk factors that would necessitate earlier screening. There are currently two options to diagnose GDM. In the one-step oral glucose screening approach, put forth by the International Association of Diabetes and Pregnancy Study Group (IADPSG) in 2010,^18^ GDM is diagnosed if, after a 75 g OGTT, any one of the criteria are met: fasting serum glucose ≥92 mg/dL, 1 h ≥180 mg/dL, or 2 h ≥152 mg/dL. Alternatively, the two-step strategy may also be employed, in which case patients are first given a 50 g nonfasting glucose load. If the 1 h plasma glucose is >140, as recommended by the American Diabetes Association, or 135 mg/dL, as recommended by American College of Obstetricians and Gynecologists for high-risk populations, patients then undergo a 100 g OGTT.^19^ Women are diagnosed with GDM if two of following glucose values are met or exceeded: fasting >95 mg/dL, >180 mg/dL, 2 h >155 mg/dL, or 3 h >140 mg/dL.

Untreated gestational diabetes increases the risk of shoulder dystocia, large for gestational age babies, and cesarean delivery.^20^ Many studies indicate that children born to mothers with diabetes mellitus are at increased risk for childhood obesity and diabetes later in life, risks independent of environment and genetics.^1^ Approximately 20–60% of women with GDM will go on to develop diabetes mellitus within 5–10 years postpartum, with about 10% being diagnosed shortly after delivery.^1^ Additionally, those with a history of GDM, and even those with impaired glucose tolerance (IGT) during pregnancy, have a higher cardiovascular risk than women who never had GDM.^21^

Given the cumbersome nature of diagnostic tests and the potential catastrophic consequences of GDM, discovering easier methods to diagnose and prognosticate the severity of GDM, including at earlier periods in pregnancy, could help improve outcomes. We set out to determine whether there was an association between GDM and various biochemical and protein markers.

### Receptor activator of nuclear factor-kappa B ligand

RANKL is a 317-amino acid peptide that leads to activation of NF-κB when bound to its receptor.^22^ RANKL is secreted by immature osteoblast-lineage cells, osteocytes, and T lymphocytes, and its primary function is the stimulation of osteoclast differentiation and activity, as well as prevention of osteoclast apoptosis.^23,24^ RANKL is regulated by various cytokines and hormones, including TNF, IL-1, IL-4, IL-6, IL-11, IL-17, glucocorticoids, vitamin D, and estrogen.^25–27^ Interestingly, associations with RANKL and activity in other tissues and with diseases other than bone are being investigated, including breast cancer and cardiovascular disease.^28,29^

In normal pregnancies, the serum level of RANKL shows variation. One study demonstrated that RANKL levels were significantly higher in the second trimester of normal pregnancy when compared with the first and third trimesters.^30^ Additionally, RANKL is essential for mammary development in pregnant mice, although it is not essential for the development of breast tissue before pregnancy.^31^ In pregnancies complicated by pre-eclampsia, one study showed soluble RANKL in peripartum and postpartum period were not different from healthy controls, but the OPG/RANKL ratio was higher due to higher levels of OPG in patients with pre-eclampsia.^32^ Another study showed a trend toward lower RANKL levels in patients with pre-eclampsia in the third trimester, but the difference did not reach statistical significance^30^

In our study, serum RANKL levels were found to be lower in women with GDM. Prior research has been conflicting, with some studies reporting no difference in RANKL in pregnant women with GDM compared with non-GDM.^12,33^ Alternatively, the prospective population-based Bruneck study showed that a high serum concentration of RANKL was a risk factor for the development of T2DM in nonpregnant humans.^34^ Animal models in nonpregnant mice with knock out of hepatocyte RANKL had improved hepatic insulin sensitivity and improved glucose tolerance when compared with wild-type mice.^34^ Additionally, activation of NF-κB (such as occurred by RANKL), has been shown to be a mediator implicated in hepatic insulin resistance and β cell loss, which would suggest that RANKL levels should have been higher in our GDM patients.^35,36^ In human and mouse islet cells, Kondegowda et al. showed that RANKL stops β cell proliferation, which can be overcome by administration of OPG. Furthermore, treatment of islets with denosumab, a monoclonal antibody against RANKL, increased β cell proliferation of human islets transplanted into rodents.^37^

It is unclear why our data in pregnant women differ from other reports in the literature of RANKL in pregnant and nonpregnant subjects. The state of pregnancy may cause changes in multiple factors which affect RANKL and other proteins, and this could explain the disagreement between our results and those in the literature looking at nonpregnant subjects.

It is possible that lower levels of RANKL in the serum of women with GDM were a *consequence* of elevated blood glucose. In patients with hyperglycemia, advanced glycation end products (AGEs) deteriorate bone quality. An *in vitro* study evaluating mouse osteocytes showed that AGEs decreased RANKL expression.^38^ However, an *in vitro* study looking at human osteoblasts found increased RANKL mRNA expression in the setting of AGEs.^39^ Additionally, other studies have shown that osteoblasts exposed to high glucose environment demonstrated no effect^40^ or increased RANKL expression, so further study is needed.^41,42^

It is also possible that serum levels of RANKL are not indicative of activity at the tissue level. Serum levels of RANKL have been found to be variable even in patients with metabolic bone disease, and currently RANKL is not used clinically as a marker of fracture risk or osteoporosis due to this variability. Studies are conflicting, showing both no difference and elevated levels in patients at high risk for fracture.^43,44^ Finally, assays used to measure RANKL in serum are imperfect. Most assays measure free RANKL, which has an unclear relationship with the amount of RANKL in the circulation bound to OPG and other serum-binding proteins.^45^

### Hepatocyte growth factor

Initially discovered in the 1980s as a protein responsible in liver regeneration, HGF is a growth factor produced by liver and placental tissues that promotes cell survival and tissue regeneration with targets found in many organs, including the liver, the pancreas, the kidneys, the heart, and the placenta.^46^ Pregnant knockout (KO) mice of the HGF receptor, c-Met, in the pancreas (PancMet KO mice), had a significant decrease in β cell mass due to decreased β cell replication as well as a premature apoptosis of β cells at gestational day 19, which correlated with the time of maximal β cell expansion in healthy pregnant mice.^11^ Blood glucose values were significantly higher in pregnant PancMet KO mice and were associated with lower plasma insulin levels. Additionally, IGT was noted before a change in β cell mass. These data suggest that HGF/c-Met has an important role in normal maternal β cell expansion and survival during pregnancy. In addition, HGF may increase insulin secretion in ways independent of the effect on β cell mass.^11^ HGF concentrations in maternal serum are below detection level before 10 weeks gestation but increase steadily after until the end of the third trimester.^47,48^ Maternal plasma HGF levels are decreased in women with small for gestational age infants^49,50^ and in severe pre-eclampsia.^51^ In nonpregnant adults, HGF is found to correlate with hyperinsulinemia in insulin resistance in obese patients with metabolic syndrome.^52^ Our study did not find a difference in serum values of HGF between patients with GDM and those with NGT. Factors contributing to the difference between our results and the literature could be that murine and human physiology are dissimilar with regard to HGF, that BMI in both our subject groups is not different, or that polymorphisms in HGF or its receptor may explain the difference, but further study is warranted.

### Prolactin

Prolactin's role in sustaining β cell mass and glucose homeostasis has been elucidated by *in vitro* and *in vivo* rodent studies. In rats, expression of the prolactin receptor on maternal islets increases during pregnancy, and treatment of islets with prolactin in culture leads to increases in insulin secretion.^53,54^ Studies in KO mice indicate that prolactin and placental lactogen are required to maintain appropriate β cell mass and glucose homeostasis during pregnancy.^55^ In humans, a study comparing plasma prolactin levels in GDM versus non-GDM individuals showed no difference between the groups during pregnancy.^56^ Similarly, we did not find a statistically significant difference in prolactin levels between GDM and non-GDM subjects. Therefore, if impaired placental lactogen signaling on β cells contributes to the GDM phenotype, it is likely due to deficits at the receptor level rather than a decline in circulating prolactin.

### Osteoprotegerin

Consistent with prior reports, we did not find a difference in serum OPG levels for subjects with and without GDM.^12^ OPG is a soluble decoy receptor that is a member of the TNF receptor superfamily. OPG binds to RANKL, thereby inhibiting osteoclastic bone resorption. Plasma levels of OPG have not been associated with bone mineral density or fractures, but have been associated with cardiovascular mortality.^57^ Previous studies have shown that OPG increases during pregnancy, followed by a rapid postpartum decline.^58^ Akinci et al. showed that women with previous GDM developing metabolic syndrome had higher OPG levels than those without metabolic syndrome and healthy controls.^59^ The Dallas Heart Study found that non-GDM was associated with higher plasma OPG levels, which was also associated with cardiovascular mortality.^60^ In our study, circulating OPG levels did not differ between the groups.

### Tumor necrosis factor-related apoptosis-inducing ligand

TRAIL induces apoptosis through interactions with death receptors.^61^ TRAIL and TRAIL receptors are expressed on the placenta and are involved in immune privilege of trophoblasts as well as trophoblastic invasion. Placental microparticles regularly are released into the circulation and therefore plasma measurements of placental proteins, such as TRAIL may be measurable in the serum.^62^ Women with hypertensive diseases in pregnancy were found to have significantly lower plasma TRAIL levels compared with women with uncomplicated pregnancies.^13^ Additionally, blocking TRAIL function in murine models of type 1 diabetes leads to increased autoimmune inflammation in the pancreatic islets.^63^ We did not find a difference in plasma TRAIL level between individuals with GDM and those without. However, investigation of TRAIL function in placental or β cell tissue may elucidate different findings.

### Tumor necrosis factor alpha

TNFα has been implicated in insulin resistance in pregnant and nonpregnant rodents and humans.^64–67^ Pregnant women without diabetes have been found to have increased levels of TNFα as pregnancy progresses.^68^ Multiple studies, including a meta-analysis have shown significantly higher levels of serum TNFα in patients with GDM.^15–17^ Correlation with circulating TNFα and insulin resistance in pregnancy has been confirmed elsewhere, and TNFα also correlates with BMI.^68,69^ Our findings did not support differences in TNFα levels in individuals with GDM, similar to another report by Georgiou et al.^70^ It is possible that we were not powered to detect differences in TNFα, or that high BMI levels and partial insulin resistance in the controls made it more difficult to detect differences.

### Plasminogen activator inhibitor type 1

PAI-1 has multiple functions, including tumorigenesis, angiogenesis, wound healing, ovulation, and regulation of anti-fibrinolytic activity of the plasma.^71,72^ Elevated plasma levels of PAI-1 have been found to be associated with insulin resistance, type 2 diabetes, and obesity.^73,74^ In 2004, Winzer et al. showed that women with previous GDM had higher serum levels of PAI-1 when compared with women with NGT during a previous pregnancy.^75^ Leipold et al. found that genetic polymorphisms of the PAI-1 gene increased the risk of gestational diabetes.^76^ In our study, women with GDM had similar plasma levels of PAI-1 when compared with controls. However, since we do not have data on genetic analysis, it is possible that genetic polymorphisms existed in some of our women with GDM.

### Limitations

There were some limitations to this study. Plasma drawn for analysis were obtained during 1-h GCT or 3-h GTT. The timing of the sample collection from time of glucose load was not controlled. It is possible that the glucose load before specimen collection or ambient glucose levels at the time of collection could have had an effect on plasma levels of the proteins that were analyzed. Additionally, there were a number of women who tested negative for GDM during their 3-h GTT, but had failed their 1-h GCT. Therefore, they were considered controls in our study, but possibly had some mild insulin resistance and glucose intolerance which may have affected the results. All blood draws occurred during weeks 24–32, so it is unknown if a difference would have been noted if drawn later or earlier in pregnancy. Additionally, white women were under-represented in our study, as most of our subjects were African American or Hispanic. Many of the other reports published in the literature have a higher representation of Caucasian subjects, making it possible that race or ethnicity impacts the cytokine/protein profile of women during pregnancy, explaining our failure to confirm the findings of other researchers. Finally, due to the cross-sectional design of the study, it is not possible to derive a causal link between RANKL levels and GDM.

## Conclusions and Future Directions

GDM can have complications to the mother and the fetus. While the exact mechanism of GDM has not been elucidated, recent studies have suggested that various serum proteins may contribute to or correlate with GDM. In our study, serum HGF, OPG, prolactin, TRAIL, TAI-1, and TNFα were similar in women with and without GDM, but RANKL levels were lower in women with GDM. Further investigation is needed to determine whether there are suitable serum markers for diagnosing GDM or determining prognosis or severity. Future work, including larger sample sizes and tissue analysis, could help elucidate whether serum RANKL levels or other proteins could be useful in this regard.

## Abbreviations Used

AGEs: advanced glycation end products
BMI: body mass index
GDM: gestational diabetes mellitus
HGF: hepatocyte growth factor
IGT: impaired glucose tolerance
KO: knockout
NGT: normal glucose tolerance
OGCT: oral glucose challenge test
OGTT: oral glucose tolerance test
OPG: osteoprotegerin
PAI-1: plasminogen activator inhibitor type 1
RANKL: receptor activator of nuclear factor-kappa B ligand
TNFα: tumor necrosis factor alpha
TRAIL: tumor necrosis factor-related apoptosis-inducing ligand
